# The Role of Hopelessness in Patients With Borderline Personality Disorder

**DOI:** 10.1097/PRA.0000000000000813

**Published:** 2024-10-03

**Authors:** Andrea Aguglia, Daniele Cioci, Matteo Meinero, Valeria Placenti, Edoardo Verrina, Davide Bianchi, Laura Fusar-Poli, Alessandra Costanza, Irene Schiavetti, Andrea Amerio, Mario Amore, Gianluca Serafini

**Affiliations:** * Department of Neuroscience, Rehabilitation, Ophthalmology, Genetics, Maternal and Child Health, Section of Psychiatry, University of Genoa, Genoa, Italy; † IRCCS Ospedale Policlinico San Martino, Genoa, Italy; ‡ Department of Mental Health and Pathological Addictions, Lavagna Local Health Authority, Lavagna, Italy; § Department of Brain and Behavioral Sciences, University of Pavia, Pavia, Italy; ∥ Department of Psychiatry, Adult Psychiatry Service (SPA), University Hospitals of Geneva (HUG), Geneva, Switzerland; ¶ Department of Psychiatry, Faculty of Biomedical Sciences, University of Italian Switzerland (USI), Lugano, Switzerland; # Department of Psychiatry, Faculty of Medicine, Geneva University (UNIGE), Geneva, Switzerland; ** Department of Health Sciences, Section of Biostatistics, University of Genoa, Italy

**Keywords:** borderline personality disorder, hopelessness, alexithymia, substance abuse, emergency psychiatry, suicide, suicide behavior, coping, sensory profile

## Abstract

**Background:**

The goal of this study was to evaluate specific characteristics associated with hopelessness, potentially correlated with coping strategies, sensory profile, and alexithymia in patients with borderline personality disorder (BPD).

**Materials and Methods:**

Two hundred twenty-four (N=224) inpatients completed a clinical interview with administration of the Beck Hopelessness Scale (BHS), the Adolescent/Adult Sensory Profile (AASP), the Coping Orientation to Problems Experienced Inventory (COPE), and the Toronto Alexithymia Scale (TAS).

**Results:**

Hopelessness was significantly associated with female gender, more hospitalizations, current suicidal ideation, number of suicide attempts, current and lifetime medication abuse, and alcohol misuse. Furthermore, patients with BHS ≥ 9 had higher scores in low registration, sensory sensitivity and sensation avoiding in AASP, higher rate of alexithymia, and the use of maladaptive coping strategies.

**Conclusions:**

Hopelessness in BPD was associated with higher severity of illness, alternative process sensory input from the environment, reduced ability to cope with stressful events, and alexithymia. Therefore, a routine assessment of hopelessness in patients with BPD could lead to better and more specific therapeutic strategies.

Borderline personality disorder (BPD) is a psychiatric disorder characterized by emotional dysregulation, impulsivity, and instability in interpersonal relationships and self-image. People suffering from BPD experience intense and sudden mood and emotional swings, severe difficulty in understanding and managing emotions, and chronic feelings of emptiness.^1^ BPD is the most common personality disorder, with a prevalence of 1% to 3.9% reported in the general population and up to 25% among psychiatric inpatients.^2^ In clinical settings, about 75% of patients diagnosed with BPD are women, although findings from community-based samples show similar prevalence rates in males and females.^3^ BPD is frequently associated with other psychiatric disorders, such as mood and anxiety disorders and substance abuse, leading to high levels of functional impairment and rates of hospitalizations, and resource utilization.^4^

Patients suffering from BPD have also an increased risk of suicide and self-harm.^5^ Indeed, suicidal behavior (SB) represents a core clinical feature of BPD, and it is listed as one of the diagnostic criteria for BPD in the *Diagnostic and Statistical Manual of Mental Disorders, Fifth Edition, Text Revision* (DSM-5-TR).^6^ A very recent review showed that 50% to 90% of patients suffering from BPD have a history of suicide attempts (SAs), with a rate of death by suicide of 3% to 10% and a 52-fold increase in the suicide rate among individuals with BPD compared with the general population.^7^ Nevertheless, not all patients with BPD share the same risk for SB, with patients with BPD and comorbid major depressive disorder, substance use disorder, and/or a history of prior SAs showing a greater risk of SB than other patients with BPD.^8,9^ Childhood trauma and antisocial traits also increase the risk for SB in BPD.^10^

Self-harming behavior also includes non-suicidal self-injury (NSSI), which can be defined as the intentional self-infliction of damage to one’s own body tissue without suicidal intent, although the presence or absence of suicidal intent is often very complex to assess in daily clinical practice. NSSI is considered a risk factor for further acts with suicidal intentionality.^11,12^ About 90% of adult patients suffering from BPD have a history of NSSI and two-thirds of them report their first presentation before the age of 18.^13^

Hopelessness, defined as an emotional state characterized by a negative outlook on the future and by feelings of powerlessness and inability to change one’s present situation, is a significant predictor of suicidality, and it is particularly related to a short-term rise in suicidal ideation (SI).^14–16^ A study comparing SB in patients diagnosed with BPD, depression, and both BPD and depression showed that hopelessness, in association with impulsivity, increased the risk of SAs and suicide in all groups (this association, particularly, could explain SA and high-lethality SA, among other different risk factors), suggesting its role as a transdiagnostic vulnerability factor for SB.^17^ Moreover, Beck and coworkers showed that hopelessness was an independent and more powerful predictor of suicidality than depression.^18^

Alexithymia is defined as an impairment in identifying and describing one’s own emotions, and several studies have reported high levels of alexithymia in patients with BPD.^19–21^ A recent study demonstrated that patients with BPD who have difficulty identifying their own emotions tend to display deficits in perceptions of facial emotions which, in turn, may lead to misperceptions of social signals and, thus, contribute to excessive emotional intensity and tension in social situations.^21^

Finally, a pivotal role in the development of various psychiatric disorders, including BPD, involves the alteration of sensory processes, often in association with maladaptive coping techniques and gender.^22–26^ Furthermore, subsets of patients with sensory sensitivity and sensation avoidance have been reported in patients with BPD.^27^

To the best of our knowledge, no studies have focused on the potential association between feelings of hopelessness and other significant clinical dimensions, including sensory profiles, coping strategies, and alexithymia in patients with a primary diagnosis of BPD. Our main hypothesis is that feelings of hopelessness in patients with BPD could be related to a higher prevalence of alexithymia, a different pattern of sensory profiles, and an altered use of coping strategies. Furthermore, we expect that hopelessness could be related to a higher prevalence of SI and SB. Consequently, our findings could also lead to a greater awareness of these issues during the clinical assessment of this category of patients and to the implementation of a new nonpharmacological treatment approach. The results of our study could assist clinicians who deal with the management of patients at higher risk for suicide every day, and they could lead to a deeper understanding of this complex issue.

Therefore, the goals of this study were (1) to evaluate specific characteristics associated with feelings of hopelessness in this psychiatric population and, (2) to investigate the potential correlation between feelings of hopelessness and coping strategies, sensory profile, and alexithymia.

## MATERIALS AND METHODS

### Study Design and Participants

In this study, 224 patients with a primary diagnosis of BPD, according to the criteria in the *Diagnostic and Statistical Manual of Mental Disorders, Fifth Edition* (DSM-5),^28^ were enrolled. Participants who were consecutively admitted to the Psychiatry Section, Department of Neuroscience, Rehabilitation, Ophthalmology, Genetics, Maternal and Child Health, University of Genoa, Italy between April 1, 2019 and December 31, 2022, were recruited for this study voluntarily, signing a written informed consent. Participants were provided with an in-depth explanation of the study objectives and procedures with the opportunity to ask questions about the goals of the study. The clinicians explained that the first part of the study included taking the patient’s medical and psychiatric history and gathering information about the patient’s sociodemographic characteristics, while the second part of the study involved the administration of several psychometric tools developed to investigate a number of specific clinical dimensions that are often not recognized and studied scientifically. Each psychometric tool was presented and explained to the patients. They were informed that the data gathered with these tools could be useful in recognizing a subgroup of patients who could potentially benefit from a specific nonpharmacological personalized treatment approach.

The psychiatric diagnosis of BPD was confirmed with the SCID-5 PD form, which was administered to all patients by senior trained psychiatrists with at least 10 years of experience in emergency psychiatry.^29^ All patients with a current severe psychiatric disorder other than BPD, according to DSM-5 criteria on the basis of interviews conducted by senior psychiatrists when they were recruited, were excluded from the study. In particular, none of the patients enrolled in the study met diagnostic criteria for clinical depression. The heterogeneity of clinical presentations among patients with BPD often reflected impulsivity, harmful behaviors, aggression, or use of illicit substances. A minority of the patients with BPD had been admitted due to chronic feelings of emptiness, a very different clinical dimension from clinical depression.

The guidelines from the Declaration of Helsinki were followed during the study,^30^ and the study was approved by the local Ethical Review Board.

### Assessment

A clinical structured interview was conducted by expert clinicians with experience working in an inpatient psychiatric setting and with patients with SB to assess and collect data on the following sociodemographic and clinical characteristics of interest: gender, current age, marital status, educational level, nationality, current and lifetime SI, number and methods of SAs, number of voluntary and involuntary hospitalizations, and current/lifetime illicit substance use and medication misuse. Furthermore, each recruited participant completed a battery of assessments, including the Adolescent/Adult Sensory Profile (AASP), Beck Hopelessness Scale (BHS), Coping Orientation to Problems Experienced Inventory (COPE), and Toronto Alexithymia Scale (TAS-20).

The AASP^31^ is a 60-item questionnaire used to measure sensory processing patterns and preferences. It includes 6 sections: taste and smell processing, movement processing, visual processing, touch processing, activity level, and auditory processing. Each section is composed of items to which the patient can answer “almost never,” “seldom,” “occasionally,” “frequently,” and “almost always.” Depending on their individual responses, people are associated with one of the following sensory profiles: low registration, sensation seeking, sensory sensitivity, and sensation avoiding. Low registration describes individuals who fail to detect sensations and who have difficulties in recognizing and expressing emotions. Each profile is assessed by 15 items as described in Dunn’s model.^32^ Furthermore, each profile is associated with a score from 5 to 75.^33^

The BHS is a 20-item self-assessment questionnaire designed to measure hopelessness. This scale evaluates 3 different dimensions: feelings about the future, loss of motivation, and expectations.^34^ The “hopelessness score” is the sum of the scores obtained from each item, with a total score ranging from 0 to 20. It is accepted as an indicator of high suicide risk for psychiatric patients and the general population. As described in the literature, a score ≥9 was found to be associated with a highly increased risk of SB.^35^

The COPE^36^ is a 60-item self-administered psychometric instrument in which participants indicate how many times a specific coping strategy is used when experiencing stressful situations that exceed their own resources to facilitate adjustment and adaptation to these kinds of events, preserving their own integrity. It is divided into 3 subscales: problem-focused coping, emotion-focused coping, and potentially maladaptive strategies.

The TAS-20^37^ is a 20-item self-report scale with a total score that ranges from 20 to 100. A score of 52 to 60 indicates possible alexithymia, whereas scores > 60 indicate the presence of alexithymia. This scale assesses the 3 core features of alexithymia: difficulty identifying feelings, externally oriented thinking, and difficulty describing feelings.

The clinical interview was conducted by and the psychometric tools were administered by expert senior psychiatrists, as described above, who were blinded to the purpose and hypotheses of this study.

### Statistical Analysis

Continuous variables are presented as means and SD and categorical variables are presented as frequencies and percentages. The Kolmogorov-Smirnov test was used to evaluate for normal distribution before statistical analyses were done.

The sample was first divided into 2 subgroups according to the BHS total score (BHS ≥9 and BHS <9), using the cut-off reported in several studies in the literature.^14,38–40^ Differences between categorical variables were evaluated by performing χ^2^ tests, while the Student *t-*test for independent samples was used to evaluate differences between continuous variables. Finally, we used a stepwise regression analysis with backward selection (ie, we started by including all candidate variables and tested how the deletion of a variable affected the statistical significance of the fit). More specifically, we deleted those variables whose removal from the model resulted in the least statistically significant deterioration of the model fit and repeated this process until no further variables could be deleted. This approach was intended to eliminate potential collinearities among different predictors of hopelessness. Finally, a correlation matrix evaluating the relationship between BHS and further variables of interest, including TAS-20, COPE, and AASP dimension T values was performed.

The Statistical Package for the Social Sciences (SPSS) for Windows 25.0 was used to perform the statistical analyses (IBM Corp., Armonk, NY). Results with *P* < 0.05 were considered statistically significant.

## RESULTS

### Sociodemographic and Clinical Characteristics in Patients With BHS Scores ≥9 Versus < 9

The total sample included 224 patients with a primary diagnosis of BPD, of which two-thirds reported feelings of hopelessness (n = 148, 66.1%). Female patients were more represented in the BHS ≥9 group compared with the BHS <9 group. A higher BHS score was also associated with more hospitalizations. The data also highlighted a link between a higher score on the BHS and current SI as well as with a higher number of previous SAs. A lifetime or current diagnosis of alcohol abuse was also reported more frequently in the BHS ≥9 group. Finally, a stronger feeling of hopelessness was linked with lifetime and current self-medication abuse. The sociodemographic and clinical characteristics are reported in Table 1.

**TABLE 1:** Sociodemographic and Clinical Characteristics According to the Presence of Hopelessness in Patients With Borderline Personality Disorder

N (%) or mean ± SD	Total sample (N = 224)	BHS ≥9 (n = 148)	BHS <9 (n = 76)	χ^2^/*t*	*P*
Sex					
Female	154 (68.8)	109 (73.6)	45 (59.2)	4.872	**0.027**
Male	70 (31.2)	39 (26.4)	31 (40.8)		
Current age in years	33.94 ± 13.50	34.28 ± 13.83	33.28 ± 12.89	−0.528	0.598
Marital status					
Single	153 (68.3)	99 (66.9)	54 (71.1)		
Married	38 (17.0)	25 (16.9)	13 (17.1)	1.123	0.772
Divorced	32 (14.3)	23 (15.5)	9 (11.8)		
Widowed	1 (0.4)	1 (0.7)	0 (0.0)		
Educational level	11.59 ± 3.31	11.61 ± 3.32	11.54 ± 3.32	−0.161	0.872
Nationality					
Italian	198 (88.4)	131 (88.5)	67 (88.2)	0.006	0.937
Others	26 (11.6)	17 (11.5)	9 (11.8)		
No. hospitalizations	3.48 ± 3.27	3.97 ± 3.60	2.53 ± 2.25	−3.195	**0.002**
Involuntary admissions	73 (32.6)	49 (33.1)	24 (31.6)	0.053	0.817
Involuntary admissions	3.04 ± 1.76	3.11 ± 2.03	3.00 ± 1.57	0.244	0.808
Current suicide ideation	116 (51.8)	88 (59.5)	28 (36.8)	10.288	**0.001**
Lifetime suicide attempts	141 (62.9)	97 (65.5)	44 (57.9)	1.259	0.262
No. suicide attempts	2.39 ± 2.45	2.70 ± 2.74	1.70 ± 1.41	−2.275	**0.024**
Alcohol abuse lifetime	135 (60.3)	97 (65.5)	38 (50.0)	5.064	**0.024**
Alcohol abuse current	86 (38.4)	67 (45.3)	19 (25.0)	8.723	**0.003**
Substance abuse lifetime	138 (61.6)	94 (63.5)	44 (57.9)	0.670	0.413
Substance abuse current	91 (40.6)	60 (40.5)	31 (40.8)	0.001	0.971
Medication abuse lifetime	87 (38.8)	72 (48.6)	15 (19.7)	17.670	**<0.001**
Medication abuse current	62 (27.7)	49 (33.1)	13 (17.1)	6.424	**0.011**

Bold type indicates statistically significant values.

BHS indicates Beck Hopelessness Scale.

### Sensory Profile and Alexithymia in Patients With BHS Scores ≥9 Versus <9

Regarding sensory profile, the data showed that the group with a positive score for hopelessness (BHS score ≥9) had higher scores on AASP low registration, sensory sensitivity, and sensation avoiding. Furthermore, hopelessness in patients with BPD was associated with a higher prevalence of alexithymia and a higher mean score on the TAS-20. These results are shown in Table 2.

**TABLE 2:** Sensory Profile and Alexithymia According to the Presence of Hopelessness in Patients With Borderline Personality Disorder

Mean ± SD	BHS ≥9 (n = 148)	BHS <9 (n = 76)	*t* Test	*P*
AASP low registration	30.98 ± 11.58	24.21 ± 9.71	−4.368	**<0.001**
AASP sensation seeking	35.35 ± 9.88	36.00 ± 12.41	0.425	0.671
AASP sensory sensitivity	38.70 ± 16.20	30.89 ± 11.27	−3.759	**<0.001**
AASP sensation avoiding	36.32 ± 10.77	31.59 ± 12.58	−2.933	**0.004**
TAS-20	64.25 ± 12.81	55.61 ± 11.32	−4.969	**<0.001**
TAS-20 ≥61, n (%)	88 (59.5)	26 (34.2)	12.809	**<0.001**

Bold type indicates statistically significant values.

AASP indicates Adolescent/Adult Sensory Profile; BHS, Beck Hopelessness Scale; TAS-20, Toronto Alexithymia Scale.

### Coping Strategies in Patients With BHS Scores ≥9 Versus <9

Regarding coping strategies measured using COPE, more pronounced differences were found on the problem-focused coping subscale. Patients with a positive score for hopelessness (BHS score ≥9) had significantly lower scores on active coping, planning, suppression of competing activities, and use of instrumental social support on the problem-focused coping subscale and on use of social-emotional support and acceptance on the emotion-focused coping subscale compared with patients without hopelessness. Moreover, hopelessness was associated with higher scores for denial strategy on the potentially maladaptive strategies subscale. Table 3 summarizes these statistical comparisons.

**TABLE 3:** Coping Strategies According to the Presence of Hopelessness in Patients With Borderline Personality Disorder

Mean ± SD	BHS ≥9 (n = 148)	BHS <9 (N = 76)	*t* Test	*P*
COPE problem-focused coping				
Active coping	10.12 ± 2.47	11.49 ± 2.55	3.876	**<0.001**
Planning	9.98 ± 2.85	10.99 ± 2.31	2.662	**0.008**
Suppression of competing activities	9.80 ± 2.55	10.79 ± 2.24	2.872	**0.004**
Restraint coping	9.13 ± 1.87	9.85 ± 2.19	1.556	0.121
Use of instrumental social support	9.52 ± 2.31	10.01 ± 2.10	3.938	**<0.001**
COPE emotion-focused coping				
Use of social-emotional support	9.53 ± 2.80	10.39 ± 2.72	2.216	**0.028**
Positive reinterpretation and growth	9.84 ± 3.99	10.59 ± 3.23	1.426	0.155
Acceptance	9.80 ± 2.68	10.72 ± 2.58	2.460	**0.015**
Humor	9.05 ± 3.15	8.91 ± 2.82	−0.341	0.734
Venting of emotions	10.59 ± 2.62	10.41 ± 3.13	−0.455	0.650
Turning to religion	8.45 ± 3.40	8.87 ± 3.79	0.846	0.398
COPE potentially disadaptive strategies				
Denial	9.69 ± 2.92	8.78 ± 2.98	−2.200	**0.029**
Behavioral disengagement	9.51 ± 2.51	9.13 ± 2.48	−1.063	0.289
Alcohol and drug disengagement	9.28 ± 3.34	9.07 ± 2.96	−0.466	0.642
Mental disengagement	9.99 ± 2.48	10.47 ± 2.35	1.396	0.164

Bold type indicates statistically significant values.

BHS indicates Beck Hopelessness Scale; COPE, Coping Orientation to Problems Experienced.

### Stepwise Logistic Forward Regression Analysis of the Results

Table 4 shows the stepwise logistic forward regression to evaluate factors independently associated with hopelessness in patients with BPD. A higher BHS score was significantly linked with a number of different sociodemographic and clinical characteristics, such as a higher number of hospitalizations, current alcohol abuse, and lifetime self-medication abuse.

**TABLE 4:** Stepwise Logistic Forward Regression to Evaluate Factors Independently Associated With Hopelessness in Patients With Borderline Personality Disorder

Variable	*P*	OR (95% CI)
No. hospitalizations	0.011	1.274 (1.058-1.535)
Current alcohol abuse	0.001	9.790 (2.465-38.878)
Lifetime medication abuse	0.001	10.387 (2.696-40.020)
AASP—low registration	<0.001	1.134 (1.059-1.214)
COPE—use of social-emotional support	<0.001	0.657 (0.567-0.831)
COPE—denial item	0.005	1.327 (1.092-1.614)
Constant	0.007	0.028

*R* ^2^ Nagelkerke=0.602

AASP indicates Adolescent/Adult Sensory Profile; COPE, Coping Orientation to Problems Experienced; OR, odds ratio.

Regarding sensory profile and coping strategies, a stronger feeling of hopelessness was positively associated with low registration on the AASP and with the denial item on the COPE, and negatively associated with the use of social-emotional support.

**FIGURE 1:**
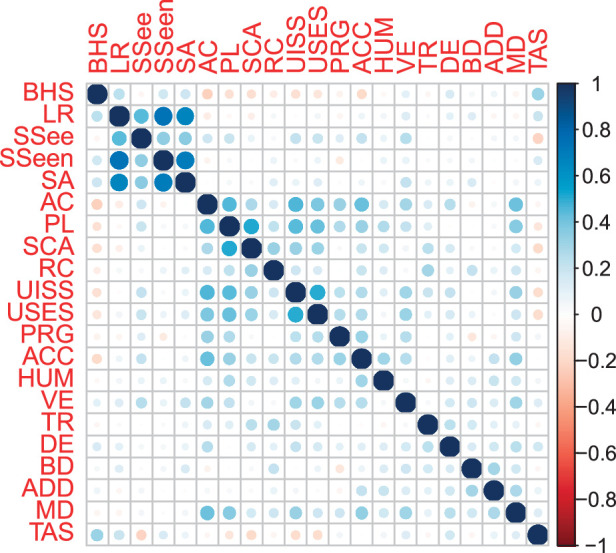
BHS, TAS, COPE, and AASP score correlation matrix. AASP indicates Adolescent/Adult Sensory Profile; AC, active coping; ACC, acceptance; ADD, alcohol and drug disengagement; BD, behavioral disengagement; BHS, Beck Hopelessness Scale; COPE, Coping Orientation to Problems Experienced; DE, denial; HUM, humor; LR, low registration; MD, mental disengagement; PL, planning; PRG, positive reinterpretation and growth; RC, restraint coping; SA, sensation avoiding; SCA, suppression of competing activities; SSee, sensation seeking; SSeen, sensory sensitivity; TAS, Toronto Alexithymia Scale; TR, turning to religion; UISS, use of instrumental social support; USES, use of social-emotional support; VE, venting of emotions.

Finally, a correlation matrix evaluating the relationships between BHS and further variables of interest, including TAS-20, COPE, and AASP dimension T values is shown in Figure 1.

## DISCUSSION

### Association Between Hopelessness and Sociodemographic and Clinical Characteristics in BPD

The aim of this study was to evaluate potential characteristics associated with feelings of hopelessness in patients with a primary diagnosis of BPD and investigate the correlations among hopelessness, coping strategies, sensory profile, and alexithymia.

Looking at the sociodemographic characteristics, most patients were female. This result could be explained by the tendency to diagnose BPD more frequently in female patients; because of different trait dimensions that are influenced by gender, treatment is more often sought by female patients with BPD than male patients with the disorder.^41,42^ A significant association between female gender and higher BHS mean score was found. This finding is consistent with results reported in a recent article in which elevated levels of hopelessness were found to be more frequently associated with female gender, a history of childhood trauma, and high levels of alexithymia.^39^ Hopelessness is considered a significant transdiagnostic clinical dimension in several psychiatric conditions, including BPD.^6^ Furthermore, a significantly higher prevalence of current SI was expressed by patients with BPD who endorsed feelings of hopelessness. This finding could be explained by the recognized and well-known role of hopelessness as an independent factor in suicidality.^18^ Finally, it would be expected that a more severe state of illness, as expressed by a stronger feeling of hopelessness, would be associated with more lifetime hospitalizations, which are often caused by a tendency to self-medicate with alcohol abuse or medication misuse. Recently, gender differences have also been demonstrated in these areas.^26^ Thus, our results may help to clarify the tendency of females with BPD to experience internalizing symptoms; providing insights and highlighting the need to monitor for self-harm and SB.^43,44^

### Association Between Sensory Processing Profiles and Hopelessness in BPD

Following Dunn’s model of sensory processing, patients with feelings of hopelessness showed higher mean scores in all sensory processing patterns, except for sensation seeking. These findings are consistent with previous scientific evidence, demonstrating the protective role of the sensation-seeking pattern against depressive symptoms and suicidality^22^, and the correlation of hopelessness with psychopathology.^23^ In our study, the low registration pattern, which describes individuals who fail to detect sensations and who have difficulties in recognizing and expressing emotions, was also independently associated with hopelessness in patients with BPD. This finding was consistent with those of another study on affective disorders in which participants with low registration responded better to antidepressant medications when they had lower hopelessness levels. Furthermore, the pattern of sensory sensitivity contributed to the prediction of hopelessness and, together with low registration, predicted changes in alexithymia levels.^24^ In our sample, no clinical depression was found and it is possible to hypothesize that low registration and hopelessness are directly associated, regardless of the presence of depressed mood.

### Association Between Alexithymia and Hopelessness in Patients With BPD

Our study found a significant association between hopelessness and alexithymia in patients suffering from BPD. A previous study demonstrated a correlation between low emotional awareness or alexithymia and borderline personality psychopathology.^45^ In particular, a stronger association with BPD was found for 2 of the main factors of alexithymia: difficulties in identifying emotions and describing them.^45^ A recent meta-analysis, based on 23 studies, showed a significant association between these specific alexithymia subscales and self-harm.^46^ From a phenomenological point of view, self-harm is used by affected individuals to manage overwhelming or poorly understood emotional experiences.^43^ Other studies have indicated that alexithymia may possibly be associated with NSSI in BPD.^47,48^ Alexithymia is also associated with a tendency to focus on negative aspects of the past and to interpret present events consequentially.^49^ This aspect could explain the negative outlook on the future, which is part of the definition of hopelessness in these patients, potentially acting as a mediator for SB. Furthermore, findings from a mini-review on the possible associations between neurological language impairment and SI/SB showed that the inability to express emotions increases suicidality.^50^ Further studies are needed to better investigate this aspect.

### Association Between Coping Strategies and Hopelessness in Patients With BPD

According to our findings, denial strategies and the limited use of social-emotional support are independently associated with hopelessness in patients affected by BPD. As a matter of fact, patients with BPD experiencing higher levels of hopelessness are less inclined to use problem-focused coping strategies. Compared with patients with BHS <9, those with hopelessness tend to be less proactive and use planning or suppression of competing activities less frequently, with consequent difficulties in approaching problems in a practical and effective way. Furthermore, these patients are less likely to use strategies aimed at regulating emotions associated with a stressful situation. More specifically, patients with BPD experiencing higher levels of hopelessness less often look for social-emotional support and have more difficulties accepting a potentially stressful situation. Finally, a tendency in these patients to disengage from stressors, particularly using denial strategies, was observed. A significant correlation between hopelessness and limited use of problem-solving coping strategies was previously demonstrated in non-psychiatric settings, for example, in oncology and neurology studies, which include clinical conditions that often have a particularly severe prognosis, but this has not previously been reported in patients with BPD.^51,52^ Regarding suicidality, lack of social support and avoidance strategies have been linked to SI, particularly in adolescents with BPD.^53^ Recently, a study demonstrated that SI is inversely related to the overall use of coping strategies, particularly those directly aimed at reducing suicidal thoughts.^54^ Thus, hopelessness may also mediate SB through the presence of maladaptive coping strategies in patients with BPD.

The findings in this study begin to provide a broader view of the psychopathology of BPD, specifically with regard to the role of hopelessness and its correlation with several main aspects of this disorder. Our results suggest the need to consider hopelessness as a pivotal clinical dimension in the phenomenology and clinical presentation of BPD. Therefore, it is important for clinicians to be able to identify this specific subtype of BPD to recognize the potential and complicated relationship between the compromised ability to manage stressful situations because of higher levels of alexithymia and difficulties in coping strategies and patients’ altered ability to describe their own emotions. The presence of hopelessness in BPD could lead to negative consequences in general patient outcomes and could potentially serve as a trigger for SB/SI.

Overall, our findings indicate that patients with BPD and feelings of hopelessness should be considered at higher risk of developing alexithymia as well as altered coping strategies and sensory profiles. Thus, these at-risk patients should be specifically screened, carefully identified, and regularly monitored during the course of illness because they could also be at risk for SI or SA or harmful behaviors. Therefore, these clinical dimensions should be considered particularly in personalizing different approaches to nonpharmacological treatment and consequently to avoid negative outcomes such as acute hospitalizations. However, prospective studies are required to further explore the impact of hopelessness on clinical outcomes in the lifetime course of illness.

Several limitations should be taken into account in interpreting our findings. First, our sample was relatively small, including only inpatients from a single psychiatric unit and considering only severe cases. In addition, the cross-sectional design of the study did not allow us to draw specific inferences regarding the temporal correlation among the investigated variables. Furthermore, patients with a hierarchically more important comorbid psychiatric disorder were excluded from the sample. The decision to exclude such comorbidity was made to avoid interferences and overlapping between psychiatric disorders such as clinical depression or bipolar disorder. However, this decision could limit the generalizability of these findings due to the exclusion of a significant number of patients with BPD and a comorbid psychiatric disorder, considering that hopelessness is a transdiagnostic clinical dimension that is often present in our patients. Our study also did not account for psychological factors that could have contributed to clinical differences between patients. Finally, we did not consider training and interrater reliability in evaluating our results.

## CONCLUSIONS

The primary aim of our study was to further explore the role of hopelessness in the pathology of BPD. A stronger feeling of hopelessness was correlated with greater severity of illness, especially considering the higher number of hospitalizations, alcohol/substance abuse, and SI or SA. Furthermore, hopelessness affected patients’ ability to cope with traumatic or stressful events and was negatively associated with the identification and recognition of emotions. Furthermore, hopelessness influenced how patients with BPD process sensory input from the environment, leading to an altered ability to detect sensations and recognize emotions, a critical aspect of borderline personality psychopathology. Given its relevance, clinicians should evaluate for the presence and severity of hopelessness when assessing patients suffering from BPD and their potential suicidal risk. These results could also lead to the use of novel therapeutic strategies. For example, alterations in coping strategies and emotional awareness may be treated with more specific interventions based on dialectical behavior therapy (DBT) techniques, avoiding off-label pharmacological treatment with antipsychotics.^55^ Currently, no specific pharmacological treatment is indicated to treat patients with BPD. However, select mood stabilizers and new antipsychotics seem to be promising therapeutic tools to treat specific clinical dimensions of BPD.^56^ There are many psychotherapies available for the treatment of people with BPD. As evidenced by a recent review,^57^ it is currently unclear which is the best nonpharmacological treatment for patients with BPD. Summarizing the main findings from that review, DBT is potentially better than the other approaches, such as cognitive behavioral therapy, client-centered therapy, and interpersonal therapy, but with only mildly encouraging efficacy reported. Finally, the preliminary results of our study suggest the need for a more in-depth analysis of coping strategies, alexithymia, and sensory profiles.
